# Involving patients in drug development for Neglected Tropical Diseases (NTDs): A qualitative study exploring and incorporating preferences of patients with cutaneous leishmaniasis into Target Product Profile development

**DOI:** 10.1371/journal.pntd.0011975

**Published:** 2024-02-21

**Authors:** María del Mar Castro, Astrid C. Erber, Byron Arana, Gláucia Cota, Claudia M. Denkinger, Nicole Harrison, Julia Kutyi, Liliana López-Carvajal, Emma Plugge, Julia Walochnik, Piero Olliaro

**Affiliations:** 1 Centro Internacional de Entrenamiento de Investigaciones Médicas (CIDEIM), Cali, Colombia; 2 Universidad Icesi, Cali, Colombia; 3 Division of Infectious Diseases and Tropical Medicine, Center of Infectious Diseases, Heidelberg University Hospital, Heidelberg, Germany; German Center of Infection Research, partner site Heidelberg; 4 Department of Epidemiology, Center for Public Health, Medical University of Vienna, Vienna, Austria; 5 Infectious Diseases Data Observatory (IDDO), Oxford, United Kingdom; 6 Drugs for Neglected Diseases Initiative (DND*i*), Geneva, Switzerland; 7 Instituto René Rachou (IRR), Fundação Oswaldo Cruz (FIOCRUZ), Minas Gerais, Brazil; 8 Division of Infectious Diseases and Tropical Medicine, Department of Medicine I, Medical University of Vienna, Austria; 9 Programa de Estudio y Control de Enfermedades Tropicales (PECET), Universidad de Antioquia, Medellín, Colombia; 10 Primary Care, Population Sciences and Medical Education, University of Southampton, Southampton, United Kingdom; 11 Institute of Specific Prophylaxis and Tropical Medicine, Center for Pathophysiology, Infectiology and Immunology, Medical University of Vienna, Vienna, Austria; 12 International Severe Acute Respiratory and Emerging Infection Consortium, Pandemic Sciences Institute, University of Oxford, Oxford, United Kingdom; Uniformed Services University: Uniformed Services University of the Health Sciences, UNITED STATES

## Abstract

**Background:**

Target Product Profiles (TPPs) are instrumental to help optimise the design and development of therapeutics, vaccines, and diagnostics – these products, in order to achieve the intended impact, should be aligned with users’ preferences and needs. However, patients are rarely involved as key stakeholders in building a TPP.

**Methodology:**

Thirty-three cutaneous leishmaniasis (CL) patients from Brazil, Colombia, and Austria, infected with New-World *Leishmania* species, were recruited using a maximum variation approach along geographic, sociodemographic and clinical criteria. Semi-structured interviews were conducted in the respective patient’s mother tongue. Transcripts, translated into English, were analysed using a framework approach. We matched disease experiences, preferences, and expectations of CL patients to a TPP developed by DND*i* (Drug for Neglected Diseases *initiative*) for CL treatment.

**Principal findings:**

Patients’ preferences regarding treatments ranged from specific efficacy and safety endpoints to direct and significant indirect costs. Respondents expressed views about trade-offs between efficacy and experienced discomfort/adverse events caused by treatment. Reasons for non-compliance, such as adverse events or geographical and availability barriers, were discussed. Considerations related to accessibility and affordability were relevant from the patients’ perspective.

**Conclusions/Significance:**

NTDs affect disadvantaged populations, often with little access to health systems. Engaging patients in designing adapted therapies could significantly contribute to the suitability of an intervention to a specific context and to compliance, by tailoring the product to the end-users’ needs. This exploratory study identified preferences in a broad international patient spectrum. It provides methodological guidance on how patients can be meaningfully involved as stakeholders in the construction of a TPP of therapeutics for NTDs. CL is used as an exemplar, but the approach can be adapted for other NTDs.

## Author summary

Our study addresses the challenge of involving patients in defining which medical product would work for their condition. When designing a new medical product, it is customary to identify a “target product profile” (TPP), which identifies the characteristics the product should have to meet in order to address the medical need it is intended for. For the product to be used as intended and achieve the desired effects, it should be adapted to the conditions and the people who will use it, so, patients’ views are important, but rarely heard.

Here, we use as an example cutaneous leishmaniasis, a skin and mucosal disease caused by a protozoan parasite, which disproportionally affects poor people across tropical and subtropical areas of the world. We collected patients’ views about product safety, efficacy, costs, treatment administration, and perceived barriers, that contribute to specifying product characteristics in the TPP.

Overall, our study contributes to the limited body of knowledge with an example and an adaptable methodology to give patients a voice in designing adapted medical products. Patients input may also contribute to redefining aspects of a TPP, such as affordability, instead of just the cost per unit. The methodology used here can be adapted and used for other neglected diseases to give patients a voice in designing medical products.

## Background

Neglected tropical diseases (NTDs) continue to burden low- and middle-income countries (LMICs) [1]. Designing therapeutics, vaccines and diagnostics for NTDs requires a deep understanding of the specific conditions in which they will be used, for them to be aligned with needs of fragile health systems and users [2,3]. Target Product Profiles (TPPs) are a valuable instrument to include these requirements and optimise the design and development of medicinal products. The Health Product Profile Directory, a dedicated directory of product profiles for health interventions containing a large number of TPPs for NTDs, was published by TDR, the Special Programme for Research and Training in Tropical Diseases [4,5].

The concept of TPPs was developed in 1997 by a Clinical Development Working Group composed of representatives from the Food and Drug Administration (FDA) and the pharmaceutical industry. It recommended using a template summing up drug labelling concepts to focus discussions and help understanding between the FDA and product developers, in addition to product design specifications [6]. There is no common format for TPPs and their use. They are mostly used either as a document signifying what a commercial sponsor would like to claim in labelling and product development [6,7], or what attributes are being sought for a public health intervention to achieve the intended health impact [8–10], in which case they are often shared with collaborators and/or made publicly available. TPPs have been indicated as useful tools for drug and diagnostic development for NTDs [11,12].

There is little methodological guidance on how to construct TPPs; actual methodologies vary. For example, PATH has constructed TPPs for diagnostics for three NTDs using comprehensive approaches involving literature reviews, surveys and interviews with experts and stakeholders, process maps and review of available diagnostic tools [13]. A TPP for a point-of-care diagnostic test for CL was developed by FIND and DND*i*, based on a draft and discussions with experts at a meeting, followed by an online survey with a larger audience of stakeholders and experts [14]. Few studies describe patients’ involvement as stakeholders in the construction of a TPP [15]. A high-level guideline [16] and a roadmap [17] have been published recently. Adepoyibi et al. [18] conducted a survey among laboratory personnel, national tuberculosis control program managers, donors, technical experts, patients and researchers, and asked them to rank the items in a TPP for tuberculosis diagnostic tools by their perceived importance. Denkinger et al. [19] included patients among stakeholders in the prioritization of TPP items for tuberculosis diagnostic tools. Studies using exploratory approaches (e.g., using qualitative semi-structured interviews or focus group discussions) of patient involvement are scarce [20].

We present a methodological and analytical approach as to involve patients in the identification of preferred characteristics of a new drug, and how this could inform TPPs. We focussed on NTDs, taking cutaneous leishmaniasis (CL) as an exemplar. CL, a parasitic vector-borne disease, disproportionally affects poor populations across tropical, subtropical and temperate regions [21]. It is caused by different *Leishmania* species, with a range of clinical manifestations. The disease results in visible lesions on exposed parts of the body, which can be distressing and discomforting, and typically leave lifelong scars. At present, there is no treatment which is effective, safe and easy to administer, supported by a robust evidence-base[22,23]. Currently, in the main endemic regions, treatment largely relies on antimonials administered intramuscularly or intravenously during 20 to 28 days, causing frequent adverse events [23]. Drug and diagnostic development efforts are ongoing mostly within public private partnerships (PDPs), such as DND*i* or FIND. American cutaneous leishmaniasis, or New-World CL (NWCL), is a form of CL caused by distinct *Leishmania* species endemic to the Americas, which has a low propensity for self-healing [24] and a risk of progression to a mucosal or mucocutaneous form (where destructive sores develop in the mucous membranes of the mouth, nose and throat) [25].

We report the methods and findings of a qualitative study assessing preferences for treatment of CL patients from Brazil, Colombia, and Austria, who were infected with New-World *Leishmania* species and received different types of treatment. This study is following an exploratory approach, that is, not restricted to pre-defined categories, or patients’ roles as end users. It complements a previous analysis where we reported patient-preferred outcomes for CL treatments, and suggested ways of considering them in the conduct of clinical trials and in clinical practice [26,27].

## Methods

### Ethics statement

#### Ethical clearance of the protocol was obtained from the following institutional review boards (IRBs) and ethics committees (ECs)

World Health Organization Research Ethics Review Committee (WHO ERC), Geneva, Switzerland. Comité Institucional de Ética de Investigación en Humanos (CIEIH), Ethics committee of the Centro Internacional de Entrenamiento e Investigaciones Médicas (CIDEIM), Cali, Colombia. Instituto René Rachou, Fundação Oswaldo Cruz (FIOCRUZ), Minas Gerais, Brazil and Comissão Nacional de Ética em Pesquisa—CONEP, Brasília, Brazil. Comité de Bioética Sede de Investigación Universitaria (CBE-SIU), Universidad de Antioquia, Medellín, Colombia. Oxford Tropical Research Committee (OxTREC), University of Oxford, Oxford, UK. Ethics Committee of the Medical University of Vienna, Vienna, Austria. Only patients above the age of consent were interviewed. Consent was obtained by signature, or an appropriate alternative, as specified by the relevant IRBs. We obtained consent from participants unable to sign by including at least one literate witness, chosen, if ever possible, by the participants themselves. Ethical procedures at each site observed the respective IRB guidance, and are further detailed in the study protocol [26].

### Study sites and populations

Using a comprehensive interview topic guide [26], individual semi-structured interviews of about one-hour duration, related to their disease experiences, preferences and expectations, were conducted with 33 CL patients at four sites in Austria, Brazil and Colombia. Under the assumption that this would cover the range of patients’ profiles and experiences, we purposively sought maximum variation along characteristics such as patients’ gender, age, treatment status (before, during, after treatment), clinical lesion presentation and causative New-World *Leishmania* species. We took the socioeconomic context into consideration by including participants from a high-income country (Austria) and low- and middle-income countries (Brazil and Colombia).

*Austria*: Three interviews were conducted at the Vienna General Hospital, Austria, which is the largest hospital in Austria and manages most CL cases imported into Austria. Austria is considered a non-endemic country for leishmaniasis, although two presumably autochthonous cases have been reported [28,29].

*Brazil*: Ten interviews were conducted at the Centro de Referência em Leishmaniose do Instituto René Rachou (CRL-IRR), Belo Horizonte, Brazil. CRL-IRR works as a reference center for management of CL for the state of Minas Gerais, in southeastern Brazil, the third Brazilian state in CL cases. Most patients were residents in small towns or rural areas within a radius of 500 kilometers from the center.

*Colombia*: Ten interviews were conducted at the CIDEIM facilities in Cali and Tumaco. The Cali facility works as a reference center for management of CL in south-west Colombia. Tumaco is a municipality located in southern Colombia, where there is endemic transmission of CL, and it is one of the areas reporting most cases of CL in Colombia. CIDEIM operates as a primary health and research facility. Patients of these two facilities are mostly civilians. Furthermore, researchers from the PECET (Program for the Study and Control of Tropical Diseases) of the University of Antioquia conducted ten interviews with soldiers who were in the Leishmaniasis Recovery Center of the national army in Boyacá. The program for management of CL for the Colombian military includes a special facility, Directly Observed Therapy (DOT) and follow-up lab tests and medical support until the end of treatment. In Colombia, the military population due to their professional duties (fight against armed groups and drug traffickers) is one of the groups most affected by CL. Interviews occurred prior to signing the peace agreement with the FARC guerrilla.

### Data collection

Interviews were conducted in the patients’ mother tongues by four researchers (MC, GC, JK, LLC) and audio recorded; interviewers took notes. Interviews from Colombia and Brazil were conducted as part of the study published by Erber et al. [26], and the Austrian interviews were conducted in 2019 following the same protocol and interview guide. Each recording was transcribed into the language in which the interview was conducted, and subsequently translated into English for analysis. Quality of the original transcripts and translations were verified by the researchers conducting the interviews.

### Data analysis

Transcripts were analysed using a framework approach [30,31], with pre-defined categories imported into Nvivo 12 (QSR International) as nodes for coding of transcripts. As a framework, we used the TPP developed by DND*i* specifically for CL treatment [32]. Two additional categories (*Perceived barriers* and *Other development needs*) were added as they emerged from the interviews and were deemed realistic to address in a broader drug development context. Two categories (*Target species* and *Stability*) were omitted as they were not addressed in the interviews. Coding of transcripts was conducted independently by two researchers (MC and ACE), and results discussed. During analysis, the coding framework was subject to continuous updating. The final coding framework is shown in **Table 1.**

**Table 1:** Coding framework. The coding framework is based on the TPP developed by DND*i* [32]. TPP categories are in bold; existing ones were amended by two added during the coding process (denoted with an asterisk *).

**1. Safety and tolerability**	**5. Treatment regimen**
1.1 Safety monitoring requirements	5.1. Administration outside of the health care facility
1.2 Tolerability	5.2. Compliance
**2. Contraindications**	**6. Duration of treatment**
**3. Efficacy**	**7. Target population**
3.1 Absence of sequelae	**8. Cost**
3.2 Complete clinical cure	8.1 Costs of products or procedures per treatment
3.3 Improved scar formation	8.2 Indirect costs
3.4 Parasitological endpoint requirement	9. Perceived barriers*
3.5 Prevention of relapse and recurrence	9.1 Availability
**4. Formulation**	9.2 Geographical barriers
4.1 Oral	9.3 Time to correct diagnosis and start of treatment
4.2 Parenteral	10. Other development needs*
4.3. Topical	

Themes, concepts or propositions that describe, help to interpret and explain aspects of the data [31] were articulated and developed by comparison between and within interviews by two researchers (MC and ACE). Themes were then assessed for potential incorporation into a TPP, and for being considered during drug development in general. In line with the exploratory nature of the study, we did not want to prioritize; therefore, we did not count the number of times a specific aspect was mentioned (frequency analysis) and did not present themes within a TPP category in a particular order.

## Results

Thirty-three cutaneous leishmaniasis (CL) patients from Brazil, Colombia, and Austria were interviewed. Most participants were male (n = 25, 76%) and the median age was 32 years (range 18–71 years). *Leishmania* species were determined either by DNA sequencing (Austria) or based on epidemiological criteria (predominant species in the region of infection; Brazil, Colombia). On average, patients had 1.7 lesions (median = 1, range 1–5). Four (12%) patients have had CL diagnosed but not started treatment, 11 (33%) were under treatment, 18 (55%) had completed their treatment; 8 (24%) had received more than one treatment (due to treatment failures or reinfection). The overview of the characteristics of the study sites and the characteristics of patients enrolled in the study are presented in **S1 Table**.

We present patient preferences along the top-level domains of the DNDi’s TPP, 1 *Safety and tolerability*, 2 *Contraindications*, 3 *Efficacy*, 4 *Formulation*, 5 *Treatment regimen*, 6 *Target population*, 7 *Cost*, and two emerging categories 8 *Perceived barriers* and 9 *Other development needs*. An overview of themes by TPP categories can be found in **S1 File**. Differences related to participants’ country, sociodemographic (e.g., income, occupation) or clinical characteristics are described when relevant. Representative quotes by TPP categories and themes can be found in **Table 2**. Trade-offs across attributes in the TPP (domains), described by the participants, are summarized in **Table 3**.

**Table 2:** Representative quotes reflecting patients’ preferences across the attributes in a TPP for leishmaniasis treatment.

**TPP Attributes and themes** [] Authors’ remarks	Representative quotes*
**1 Safety/tolerability**	
Safety monitoring requirements	*Back then I didn’t want to [be hospitalized] because I had the feeling it was more relaxed when you can go home every evening. But thinking back, I am now of the opinion it might have been better just to stay there. […] Often, I didn’t know in the evening at home how I should lie down, because my kidneys hurt, and I was very nauseous. I was given Zydis to take home, but sometimes it wasn’t enough. Then I had taken too much already and didn’t want to take any more. Then my ankle hurt again so much, then I looked at the clock, counting hours until I had to go to the hospital. 3 weeks long, that was a bit exhausting. (AT03)*
Tolerability	*Well, at times I feel a bit scared [of the treatment], but I would not back down, I am going to continue, because I got in, and I am going to finish it. Besides it is the only way to get cured. [I fear] too many shots. So many injections, and the reaction is really strong. [. . .] Well, right now I would say [as a message to people with leishmaniasis] it is best to use the medication even if it’s uncomfortable and painful. (CC09)* *If we could choose to not have side effects, it would be ideal, right? The problem is that it is part of the treatment … (BR09)* *[When they told me I had leishmaniasis] I thought "once again that drug that fucks you up". It appears that drug is toxic and leaves you sequels. (CP08)*
Description of AEs	*The treatment for me [intralesional Glucantime] was super cool. I had imagined that I could have side effects, but it was very good. I didn’t feel anything. Only the days of the infiltrations I had some swelling, which sometimes bothered me a little. (BR03)*
Fears about (second) treatment due to AEs	*That thing it’s so strong. . . Just look at the drug they are injecting you. It’s like a poison. It seems to be a common pimple, but what they inject you for that pimple it’s what hits you hard. That’s what screws you up. The cure itself fucks you up from the inside. (CP02)*
Preference for lower volume of drug	*If there isn’t another [treatment], I would take this one. I think the dosage is too much for one person though. Maybe if during the day the amount of drug is less, I think the body would assimilate it better. (CP06)*
Trade-off: Cure vs. tolerability	*To tell you the truth, I really don’t know [anything I am afraid of in terms of treatment], as long as I get cured [. . .]. No, I’m not scared about any risk. (CC08)*
**2 Contraindications**	
Reasons for contraindication	*I had had few months before a cardiac diagnosis (arrhythmia) and I was submitted to a heart ablation. Despite having been cured of the arrhythmia by ablation, the leishmaniasis treatment was considered more complicated and I was hospitalized. […] Yes [I received amphotericin B]. . . they were afraid to give me another treatment, the first line treatment, because of my previous heart condition. But today I know it should have been different. (BR01)*
Self-care	*I think not everyone has the same capacity of process the medicine. There are weaker people and so. But [leishmaniasis] can also appear again because the person doesn’t take care of himself/herself, so the treatment doesn’t work as it should. […] My nephew was one of those who smoked during treatment, so the disease got worse. I think if you drink, smoke, stay up late and do the things you should not, then that’s bad. […] That’s why I’m taking care of myself a lot. (CP10)*
Shared decision making	*[…] As I lost contact with the health promoter from there [where I live], my sister and my son told me that it was better to have the treatment here, in case there were some kind of adverse reactions to the medicine. So, the doctor told me what we could do and we agreed on a health center in near La Nave [a location in Cali, Colombia]. (CC09)*
**3 Efficacy**	
Absence of sequelae	*[I think in the future I will have] maybe heart problems. Sometimes you hear that you can have problems because of the crazy amount of medicine you received. [I am afraid of] not having the same strength again. […] You make physical effort and the body doesn’t respond the same way. (CP06)* *Scars do not bother me much. But […] the doctor told me that the drug was strong […], what if that leaves me with the pain, what if it will not stop? That’s why I do not want that the treatment be extended further. (CC09)*
Trade-off: Cure vs risks related to treatment	*Yes, I do [still want to be treated, even thinking that the lesion does not threaten your life and the remedy has risks]. […] [I could not keep this lesion, but] I would look for a treatment every way. (BR08)*
Trade-off: Cure vs. scars/aesthetic results	*Look, the first thing I asked the doctor: " Will I have a scar?" He said "probably you will get a mark, a colour change in the skin" "But will it heal?" "Yes, it will". I’m going to travel on vacation in few days, and I was worried, because I’m planning to go to the beach and to use a bikini. Then my husband asked me: "Will you find a wider bikini or something like that?” and I never had to think about that …. […] The most important thing is that I am healed. […] If you can live with another scar caused by another injury, why not? (BR01)*
**4 Formulation**	
Oral	*The one I’m taking now that is oral, it’s less traumatic than the first one I had. [. . .] This one suits me better, I mean, I feel much more comfortable. [. . .] Because you don’t get sick leave, I mean, you have to continue working somehow, and every day you have to go to get an injection, that is traumatizing (laughs). (CC01)*
Parenteral	*In my case, when I came here for the treatment, I did not sleep thinking about the injections. [. . .] I was up all-night thinking about the injections. It was horrible. […] It is the only thing I could think about. (CC08)* *I had a little trauma in my childhood–I have fear of injection. I complain because I had a surgery years ago when I saw the entire procedure, I wasn’t anesthetized properly. […] For me, on a scale of one to ten, it’s ten. [. . .] But I’m not afraid of the medicine, the drug itself. (BR05)*
Perceived efficacy of parenteral administration	*I’ve always preferred injection. [. . .] I like it. [. . .] I think injection produces a faster effect. [. . .] It’s painful, but it’s better (BR06)* *Pills for me don’t work. […] Every time I feel sick I go and say what I have so they can give me injections. I think that’s the best thing you can have, because it goes directly through the bloodstream and starts killing the viruses. [. . .] For leishmaniasis I think the injection is the best. [. . .] [I think an injection in the wound] would be good. I guess you would receive fewer doses. Also, stronger! So it works faster. (CP10)*
Local vs. systemic administration of parenteral treatment	*With the second, it was worse [regarding symptoms]. [. . .] I was very feverish and my heartbeat was fast. [. . .] [I think this is] maybe because of the crazy amount of medication they inject you. [. . .] For me intravenous would be good [as the ideal treatment]. You suffer less. (CP03)* *For me [the ideal treatment] wouldn’t be applied in the buttocks. For me it would be appliable as a serum (the patient used the term ‘suero’, a generic term for intravenous infusions–author). […] 20 syringes are a torture. [. . .] I was constantly affected psychologically because I always thought I was going to get injected in the same place, and the scar was going to remain where I had the wound. [. . .] If by any chance I get leishmaniasis again, I’m quitting the job. […] All those injections. […] It leaves you marked for your whole life. (CP04).*
Trade-off: Pain due to injections vs. perceived disease severity	*[The ideal treatment would be] pills, creams or something not based on syringes. [. . .] The injections are very strong. [. . .] You have to go through all the needles for such an insignificant thing. (CP09)*
Lack of alternatives: Injections as only treatment option	*The treatment is already okay, because there is no other way but the injections. (CP02)*
Topical	*Well, [I think the best treatment to cure Leishmaniasis would be] something you just put there. [. . .] That easy. Something you smear it on and no more. [. . .] I was told that if one does not act upon right away, it may go through the bloodstream and reach the liver. (CC08)*
Preference for creams	*[. . .] Hopefully they develop a cream that one could put on. But I have a question, why so? Why is the drug for the body [administered via injection,] knowing that one has the lesion on the skin, and the parasite is supposed to be there? (CC08)*
Thermotherapy	*[As I have access to scientific publications, I did some research and noted] that heat treatment is a standard low-cost treatment. [. . .] Actually, heat treatment helped me best, but it was never mentioned that it could be tried. [. . .] I would advise everyone to try a heat therapy themselves if it’s not being offered to them. (AT04)*
**5 Treatment regimen**	
Optimal treatment duration	*The shorter [the treatment duration], the better. [. . .] You can recover faster and you can be completely healthy. [. . .] (CP07)*
Trade-off: Treatment duration vs. cure	*It is a long treatment. . . but the important thing is to be cured. [. . .] Absolutely. (BR06)*
Administration of therapy outside of treatment facility due to long treatment duration	*I had to stay about fifteen days here, I got about 20 or 30 [injections here in Tumaco]. And the others I took home with me. Because in the countryside is where we have our farm, and we just couldn’t abandon it. [. . .] [The doctor] told me, that once I got the shots I (couldn’t) keep working, but I just can’t stop working. [. . .] A cousin gave me the injections, [. . .] in Gualao. [. . .] Here in Tumaco, a sister-in-law gave me the shots. She is a nurse. (CC03)*
Recovery time after treatment [Before release to combat area (soldiers)]	*Because the soldier finishes the treatment and is sent back to the Battalion right away. […] Or they leave you here if you’re not good enough. […] So, for me, some recovery days would be okay. Like that, the wound can recover well. (CP02)*
Compliance [Fear of disease progression]	*The treatment is the only thing that can cure leishmaniasis. [. . .] [If I decide not to have treatment] the wound keeps growing and growing and it will be my problem because I decided not to get the treatment. So, when the wound gets bigger, I will have to repeat the treatment like other partners that finish the first treatment with Glucantime and it didn’t cure them because the wound is too big. (CP08)*
Trade-off: Compliance vs. side effects	*[I received the injections] into the vein [. . .] every day, I was tired, it was painful. . . I had to come here every week, I had to have my blood collected, the other morning I had to collect blood again and receive the medication in my town. . . it was painful, yes, but I did everything correctly. (BR06)*
Low compliance due to fear of injections	*Yes, really, I have [been compliant] with the pills, I have been taking them, she prescribed three daily; but with the injections, I was not. (CC07)*
Treatment interruptions due to AEs	*[They sent me 60 injections and I received 48] because] I couldn’t tolerate it anymore, I couldn’t even sit down, nothing. [. . .] So I rested for three days and then started again; so that’s why I think the treatment didn’t do me good because later, […] it came back. (CC07)* *I went in and then I received the first Pentacarinat. Then I got problems with my blood glucose, extremely low blood glucose levels, there I wasn’t well. […] And then I think [I took] 2 days of break and one more infusion, 3 days break, one more infusion, about that. . .perhaps about one week or a little more in the hospital. (AT03)*
Treatment frequency	*If we had an oral medication to treat, a faster treatment, it would be great, because there is a certain disorder in going daily to receive medication. If there was something to take home it would be better, it would be easier too. (BR01)*
Trade-off: Place of administration vs. treatment frequency	*[When doctors were discussing treatment options with me,] they asked what would be my preference: come here to receive the infiltration once a week or receive the medicine every day in my town. I decided to receive the remedy here, […] because once a week is much easier. [. . .] It was a blessing. Since the day I got here, my lesion is just improving! (BR08)*
**6 Target population**–Quotes and detailed description are available in **S2 File**	
**7 Cost**	
Administration of injections	*I had to pay to my cousin [to get the injection]; my sister-in-law did not charge me anything. But my cousin, I had to pay her, 20,000 pesos. [. . .] She told me to give her whatever I felt like. So, I gave her 20,000 pesos. (CC03)*
Consultation fees	*In my town there is no infectious disease specialist. [. . .] I mean, there are several physicians on the private network, but considering my financial condition I couldn’t afford it. I would have to wait until it was scheduled by SUS [Sistema Único de Saúde, the Brazilian public health system], in Belo Horizonte. It usually takes too long. (BR05)*
Transport costs	*Ah, it takes 1 hour [. . .] by canoe [to reach] Tumaco. By boat [with motor] 20 or 30 minutes, but sometimes it’s hard, because sometimes you do not get a boat or a canoe to come. I have my own [aquatic] vehicle to come if there is an emergency, but [sometimes is not possible to cover the costs by transporting passengers or goods] and one uses a lot of gasoline […], so it is better to pay the 15,000 pesos as a passenger because if not, you buy 100,000 pesos of gasoline for a round trip. Things are so expensive. (CC03)* *It was tough arriving here. I had to pawn my cell phone, I had to arrive to my sister’s and then she lent me money. [. . .] No help at all from the army, despite the fact that I was working there. They took me out, they gave me their permission and that’s it. [. . .] Only my brother-in-law lent me the money for these tickets to come here. (CP02)*
Profession-related costs (inability to work, or change of occupation)	*My payment is based on days of work. [. . .] I want to do my treatment. [. . .]. No work, no money. I’m pleased to get here and do the treatment. [. . .] [It is] impairing my income, [. . .] [but] No, it doesn’t bother me. [. . .] I’m [coming on my own] [. . .], by bus. [. . .] I paid the ticket. [. . .] (BR08)* *I was unemployed [when symptoms started]. I’ve been looking for a job the last months. [. . .] Several employers told me that [they couldn’t hire me because of the wound, an ulcer in a visible area on the arm]. They told me directly [that this was the reason]. [. . .] [This happened] twice. [. . .] After that, I gave up, I said "there is no way". [. . .] My concern is [not about the disease or the possibility of future complications, but] about work. (BR02)*
Having to stay away from home for treatment	*I live with a family member, I live there and thank god, they have helped me. [. . .] Right now I’m not doing anything [work-wise], because I work with leather, I make belts and that kind of things for farm animals. But not right now. (CC09)*
**8 Perceived barriers**–Quotes and detailed description are available in **S2 File**	
**9 Other development needs**	
Investments in research	*I think we must invest more in research, to improve things. Because I know, in Brazil, at least based on the information that I had, we have just one medicine and that this treatment has many risks. (BR05)* *You should keep on researching about the cream. Hopefully soon it becomes more effective than the injections or maybe it can help heal quicker, that would be ideal. (CP01)* *I would like that for leishmaniasis there was something like a vaccine. […] For example in my case, I go through this treatment now, I spend a lot of time and all that stuff, effort; and sometimes one has to be in those areas where the disease remains […], and what if one gets infected again, then he would have to go through the same treatment again. (CC09)*
Information and dissemination activities	*[I would provide] information, more dissemination of information about the disease. You need to explain to people how the disease is transmitted, that there is treatment and people need to be seen by a doctor, […] they shouldn’t think it is a normal wound. (BR05)*

* Patients’ unique identifiers contain a two-letter code corresponding to the study site (AT-Austria, BR-Brazil, CC-Colombia/CIDEIM, CP-Colombia/PECET).

**Table 3:** Identified trade-offs. Six trade-off pairs could be identified. Among these, three were risks traded off with cure.

Aspect 1	Aspect 2
Cure	Risks related to treatment
	Scars/aesthetic results
	Long treatment duration
Pain due to injection	Perceived disease severity
Compliance	Side effects
Place of administration	Treatment frequency

### 1 Safety/Tolerability

Patients described their experience with the types, timing and sequence of tests required to monitor the treatment safety. These descriptions were most detailed for the Colombian soldier population, the majority of them treated with systemic meglumine antimoniate. Information on preferences of monitoring was limited. Among these, two patients mentioned how monitoring needs determine the treatment location: One Colombian patient decided to stay in a city during the treatment to have better monitoring of the treatment, while one Austrian patient preferred to be treated in a hospital instead of on an outpatient basis, due to the severity of the experienced side effects. When speaking about systemic antimonials, some patients preferred a lower volume of drug to be administered due to the pain and adverse events (AEs) experienced.

Patients’ descriptions of multiple adverse events in response to pentavalent antimony, pentamidine and miltefosine are in line with the literature. Many discussed tolerability of treatment and reported adverse events (AEs), at times in an emotional manner, describing the often-complex considerations they face. Patients often saw AEs as an inevitable part of the treatment, and accepted them. Fears of receiving treatment due to the injections and the side effects were illustrated by two Colombian soldiers. They met others requiring more than one course of antileishmanial treatment and were afraid of receiving a second treatment and its potential consequences (“*it seems like a common pimple*, *but what they inject you for that pimple it’s what hits you hard" -CP02-)*.

In contrast, three Brazilian patients described their positive experiences with intralesional administration of antimonials. Trade-offs made are reflected in how patients chose to ‘ignore’ the risks associated to the treatment when weighted against the need of being cured.

### 2 Contraindications

Patients described the reasons for which the systemic antimonial treatment was contraindicated, including hypertension and arrhythmia, and alternative therapies offered to them. Some participants, mainly soldiers, highlighted the need for self-care or ‘taking care’, such as avoiding certain food or activities, to contribute to the healing process. Notably, one Brazilian patient with contraindications described moments of shared decision making with the treating medical team, which was not observed in other groups. One Colombian patient described discussing the location of treatment, based on the need of need of monitoring, with his family.

### 3 Efficacy

Treatment efficacy was discussed extensively by patients. Efficacy was understood as clinical cure in relation to the lesion, the absence of sequelae, scar formation and the prevention of disease relapse/reinfection. As part of a larger study on outcomes, a number of aspects were already reported on in a separate publication [27]. In this category, we identified efficacy as an important driver in the trade-offs made about treatment (Table 3). Patients discussed the preference for an effective treatment in healing the wound, regardless of scar appearance, potential side effects or treatment duration.

As described in *Tolerability*, part of the fear of disease relapse and reinfection was having to repeat the treatment. Some patients were aware and afraid of the risk of reinfection when living in an endemic area. Thus, long-term sequelae of the treatment, particularly antimonials, and fears of sequelae related to the disease itself, such as disease progression, could be avoided by successful treatment.

### 4 Formulation

In general, patients preferred oral and topical treatments due to the easier administration and being perceived as ‘fitting’ the disease. This conceptually interesting discussion was mentioned by different patients, who expressed that, since the infection is local, they would prefer the treatment to be also applied locally: a topical treatment for a local infection. This in addition to the perceived contrast between the treatment and the perceived disease severity, i.e., a long parenteral treatment for a ‘pimple’. By some patients, oral formulations are seen as easier, and an ideal treatment when compared to systemic antimonials.

Most Colombian patients, both from the military and the civilian population, had a preference for ointments over injections. One mentioned the potential timeliness of applying a cream to the wound, assuming than an early treatment may limit the infection. Physical therapy in the form of thermotherapy was only mentioned by Austrian patients.

When discussing parenteral formulations, patients often reported ‘fear of needles’—an aversion, or even a trauma, against injections. In contrast, some patients perceived injections as working faster and more effective than alternative modes of administration. While others, such as two Colombian patients, reported injections as the only treatment option. Mainly Colombian military patients discussed localized (directly on the lesion) versus systemic administration of antimonials. In this context, some patients prefer intravenous infusion (against intramuscular) due to the volume of medication, and less pain. However, intralesional injections were seen as preferable by being ‘stronger’, faster, and requiring fewer doses. This highlights the trade-offs between efficacy and other product characteristics.

### 5 Treatment regimen

*Treatment regimen* was found to be a TPP category rich in themes and trade-offs. Patients perceived long treatment durations as burdensome, particularly against the need to balance work obligations, and in connection with indirect costs (days of work lost). These were more marked for those living in remote areas. Twenty days to one month were seen as acceptable by some patients, corresponding to their experience, although they prefer the faster option to heal the skin lesion. In contrast, two patients at the military treatment center wanted additional recovery time before being sent back to operations. Patients described alternatives to mitigate the impact of the long treatment, including seeking help from others, often relatives, to administer the drug at or near home. Long treatments were accepted as a trade-off to achieving clinical cure.

The theme of compliance was central. In general, being treated, and taking the full treatment course, was seen as important to avoid relapse and sequelae. Many respondents continued treatment despite side effects. Reasons for this were the fear of disease progression and its consequences. Other patients reported treatment being paused, or suspended, due to adverse events, thus prolonging the overall treatment duration.

Regarding treatment frequency, daily administration of parenteral drugs was considered as too frequent, and patients preferred weekly injections. This was partly related to the logistics of daily parenteral drugs, for example, having a different health provider during the weekends at the hospital, which may not be familiar with the case or the drug. In contrast, daily administration of oral treatment or a drug that can be used at home, was accepted.

The availability of the drug or trained healthcare providers also influenced the decisions of where to receive treatment. When facing this situation, a Brazilian patient opted for the weekly injections in another city instead of daily ones near their residence.

### 6 Target population

Not all respondents addressed this category; often interviewers were being asked to clarify. Most agreed that everyone should be treated, for reasons such as an intrinsic right to treatment, patients’ quality of life, or to avoid disease progression. A few patients noted that special populations, such as children, should not be treated due to the pain and frequent adverse events of the medication. Findings are available in more detail in **S2 File**, as they were difficult to interpret and incorporate in a TPP.

### 7 Cost

Costs were frequently mentioned by patients, especially those related to procedures such as administration of injections, consultations, diagnostic tests, and transport. Inadequate or over-the-counter medications before CL diagnosis were also discussed, but rarely costs related to antileishmanial drugs, in line with the treatment being provided for free in Austria, Brazil and Colombia. One Colombian patient mentioned paying for administration of the injections outside of the health care system. Costs of diagnostic and pre-treatment tests required to define eligibility to systemic antileishmanial drugs (e.g., blood cell count) were sometimes paid by patients to expedite the initiation of treatment.

In a high-income setting, an Austrian patient describes paying a 140 EUR (154 USD) fee for specialized care to shorten waiting times, while a Brazilian patient described the challenges trying to access specialized care in their town, both due to the costs of the appointment and limited of availability of specialists. Some patients, particularly among those living in remote areas, reported significant transport costs to treatment facilities using a variety of means of transport, as well as time lost due to the journey. One patient describes pawning and borrowing money to afford transportation costs. This aspect is closely related to *Geographical barriers* (**S2 File**).

Indirect costs were mainly related to days of work lost and were mentioned as significant. Taking time off during treatment without payment was considered difficult for patients who worked as day laborers, or self-employed, some were not able to work at all during treatment, or not able to find work. Having to stay away from home for treatment was another reason for indirect costs and often affected patients’ income prospects. This contrasted with patients with formal employment, such as the Colombian soldiers or the Austrian patients. One Austrian received a sick leave of 4–5 weeks, noting that she was lucky and was not afraid of losing her job, due to her employer being treated for CL at the same time.

### 8 Perceived barriers

Patients reported barriers including geographical location, availability of treatment, migratory status or occupation (for example, civilian patients from Colombia reported difficult or no access to CL treatment for patients employed in illegal professions, or migrant workers) **S2 File**. Often there was a combination of different barriers that resulted in indirect costs and delayed initiation of treatment.

### 9 Other development needs

Patients emphasized the need for investments in research, to develop alternative drugs with a better safety profile. In addition, some patients emphasized the need to invest in vaccine development, knowing the risk of reinfection for people living in endemic areas.

In addition, patients emphasized the need for general information and dissemination activities, including information about the disease, such as the mode of transmission and treatment options, as well as campaigns to de-stigmatize the disease.

## Discussion

By collecting the perceptions, values, and preferences of a range of patients regarding product characteristics for cutaneous leishmaniasis treatment, this study provides a pathway to integrating patients’ perspectives in the design and development of TPPs for novel treatments. Overall, our findings show that the treatment preferences of a broad spectrum of CL patients fitted well to a framework comprising the categories described in DND*i*’s TPP [32]. This was particularly evident for *Safety and tolerability*, *Efficacy*, *Formulation*, *Treatment regimen*, and *Costs*, which yielded rich findings, and less for *Contraindications*, where patients mostly stated experiences. Preferences for *Target population* were difficult to identify, thus, along with *Target species* and *Stability*, might be less suitable or would require a modified approach due to their rather technical nature. Two additional categories, *Perceived barriers* and *Other development needs*, were identified. Often, preferences connected several categories, e.g., via trade-offs.

Efficacy was a central topic, discussed extensively by patients [27] and often assessed in relation to experienced discomfort, or as part of trade-offs influencing treatment decisions. Impact of scars was mentioned, but social stigmatization, including gender-specific, was described to a lesser extent than patients suffering from old-world CL forms, likely indicating a cultural component [27,33–38]. Findings about safety and tolerability were closely related to experiences following treatment, with adverse events similar to those reported in clinical trials and observational studies for NWCL treatments [22,23]. Fears of sequelae and relapses motivated patients to complete the full treatment regimens, despite adverse events and burden associated with the long duration of treatment.

Duration of treatment was related to the category of formulation. Parenteral treatment once-a-day was considered as too frequent, while daily administration of oral treatment or a drug that can be used at home was accepted. In general, patients preferred oral and topical formulations, in line with WHO’s road map for neglected tropical diseases 2021–2030, which proposes development and scale-up of an easy to administer oral or topical treatment that could be used in health centres as a critical action for CL [39]. At the same time, parenteral administration (infusion and injection) was perceived as more efficacious by some patients. This relates to reported perceptions of injections as more effective [40] and act faster, relieve symptoms quicker and involve less risks than oral drugs [41]. Notably, parenteral meglumine antimoniate was the most widely used treatment in this study population. As with contraindications, the lack of shared decisions in case management was apparent, with interviewees seeing injections as the only option to get cured.

Costs were frequently mentioned and widely discussed by patients. Loss of work and income contrasted between day laborers and those with formal employment (e.g., military) and between Latin America and Austrian patients. Indirect costs have been identified as an important part of the economic burden of leishmaniasis in Asia [42]. They compounded with perceived and encountered barriers (**S2 File**), reflecting the often-complex life realities of patients. This was most pronounced in the interviews with patients living in very remote areas, migrant agricultural workers and those working with illicit crops, which is aligned with experiences in rural areas of Colombia [43] and Latin America [44]. NTDs are diseases of poverty [45]. These findings are in line with studies addressing the considerable socioeconomic impact of CL [35,46], and reflect the need to expand the attribute of costs of a product to its affordability. Whereas a target product price is often included in a TPP, affordability to end-users is not [5] and accessibility is only included in a limited number of TPPs [5].

Findings related to the target population were difficult to interpret, probably because the question ‘Who should be treated?’ was poorly understood. This could be improved e.g., by providing explanations, or moderated focus group discussions (FGDs) to allow for clarifications in a group context. As we only interviewed patients who wanted to be treated, this is likely reflected in their opinions.

### Incorporation of patients’ preferences in the TPP process

Central among the barriers to involving patients in drug development is the lack of methodological guidance, as reported by drug developers, patients and patient advocates, regulators and funders [47,48]. Recent initiatives have addressed this at a general level [16,17], and our study provides methodological guidance using an open, exploratory approach taking an NTD as an example. We designed and published an interview topic guide that allows an in-depth exploration of experiences and preferences [26], and reflections upon these, instead of focusing on TPP categories *a priori*. This interview topic guide could be adapted for similar diseases, in particular skin NTDs. Similarly, as done in this study with DND*i’*s TPP, a coding framework for analysis could be adapted from existing TPPs for any particular disease or condition of interest, or guidance documents [6].

We thus advocate for incorporating patient preferences during technical discussions with experts and stakeholders when a TPP is being initially constructed, and then during revisions (often happening at regular intervals, or as required) [5]. This could inform the definition of ideal, acceptable or minimal requirements for each category, which very often feature in TPPs [6], in addition to parameters such as safety or efficacy endpoints which could, in turn, be considered in clinical studies. We posit that exploring patients’ perspectives would add value by bringing up aspects not otherwise considered by stakeholders and may also guide the R&D agenda. For example, the findings reported under other development needs highlight the importance of research into better treatments and a vaccine for CL [49].

Findings could be consolidated by quantitative instruments focusing on selected aspects of interest; identified themes could directly inform questionnaire design. A survey on preferred formulations pre- and post-treatment among patients has been shown to complement clinical trials of CL treatments [50]. The trade-offs identified in our study, and more general, identified risks and benefits could be further investigated using a quantitative discrete-choice-experiment (DCE) or triadic comparisons [51]. DCEs, used in healthcare research [52,53], have been used previously to inform drug development [54–56]. Previous studies have successfully used an initial qualitative phase in order to inform a subsequent discrete-choice experiment [57]. This could be complemented by consultations with drug developers to gain insights into the construction and updating of a TPP, as well as to identify stages where patient input could be useful.

As suggested by others [5,17], considerations related to accessibility in combination with the closely related concept of affordability (as represented by the categories *Cost* and *Perceived barriers)* should be embedded within a TPP, in particular in any prospective profile describing preferred product characteristics. Both ideal and acceptable targets for direct and, if possible, indirect costs should be included. Compliance is seen as a central issue across themes, and could be addressed meaningfully; first, by incorporating patients’ preferences in drug development in general and second, by addressing the actual and potential reasons for non-compliance that were brought up, such as experienced adverse events or geographical and availability barriers.

### Generalizability and transferability of findings

As this is a qualitative study using a non-probability sample [58], it is therefore not likely to be representative of the entire spectrum of patients across regions. In our study, we paid attention to the transferability of methods, and, to a certain extent, of findings by clearly laying out settings (see *Study sites and populations* in the *Methods* section) and limitations (see *Strengths and limitations of the study*). Furthermore, we found the different settings reflected in the preferences, such as the civilian vs. the military population in Colombia, and a limited number of patients from a high-income, non-endemic area.

We did not include patients suffering from Old-World CL (OWCL) forms, and concentrated our efforts on NWCL forms, by a distinctive *Leishmania* species spectrum endemic to the Americas with very limited self-healing, and often progression to a mucosal or mucocutaneous form [24,25].

### Strengths and limitations of the study

Interviews were conducted in patients’ mother tongues, including German, Portuguese, and Spanish. The analysis of pooled interviews transcripts, translated into English, was performed independently by two researchers, and informed by notes taken during the interviews as well as discussions with the interviewers; this process ensured consistency while taking the cultural context into consideration.

Instead of designing a topic guide focussing on TPP categories *a priori* and asking for preferences, we chose a broad topic guide to allow for an in-depth exploration of experiences, and reflections upon these. Using such a comprehensive interview guide, other aspects of the lives of patients, such as the impact of disease and treatment on everyday life, or disease-specific topics such as stigmatization due to disfigurement and scars, could be explored as well.

We only interviewed patients with NWCL who had sought, started, or completed their treatments and whom we were able to follow up. Hence, by design we are only able to report on patients’ experiences with therapies, which may influence their perceived ideal and acceptable treatments. In addition, each participating country has marginalized populations disproportionally affected by CL, e.g., refugees or migrant workers, which were found to be difficult to be followed up and reluctant to be interviewed (JK, MCN, personal communication). Finally, we used medical terminology instead of patients’ own words, in line with the aims and existing literature. We are aware that this might compromise presenting the richness of patients’ experiences.

### Further research

Future studies could try to include CL patients who were lost to follow-up in trials or routine treatments, or marginalized populations (e.g., immigrants, migrant workers). Methods such as a discrete-choice-experiment (DCE) or triadic comparisons, as outlined above, could provide a quantitative assessment of identified trade-offs, and contribute to the generalizability of results.

The methodology is designed so that it could be adapted for other NTDs, in particular other skin NTDs [59]. Modifications to the sampling strategy, the interview topic guide and, if necessary, the analysis framework (which could be based on an existing treatment TPP) would allow for consideration of the specific context, including patients’ social and cultural circumstances.

## Conclusions

Addressing NTDs will require innovative approaches [60] and this study contributes to the diverse efforts required to tackle these diseases effectively. We were able to show that patients can be meaningfully engaged in the construction of TPPs, and demonstrate the feasibility of a methodology involving an international patient population of an NTD. We recommend that Access and Affordability, directly informed by patients’ experiences and preferences, be included as separate categories in any TPP. Patient involvement is critical in the development of any TPP, and funding should be set aside to facilitate meaningful involvement.

### Contributors

ACE, BA, EP, GC, LLC, MC and PO conceived of and designed the study, and developed study instruments. MC, JK, GC, MC and LLC collected data. EP, JW, NH and PO supervised data collection and analysis. ACE and MC coded the data, conducted the analysis, and wrote the first draft of the manuscript. All authors contributed to the manuscript and had final responsibility for the decision to submit for publication.

### Data sharing

All relevant data are within the paper and its Supporting Information files. The study protocol is published [26].

## Supporting information

S1 Table Study sites and patients enrolled in the study. (PDF)

S1 File Overview of themes by TPP categories. (PDF)

S2 File Additional findings. (PDF)
