# Supplementary material for: Involving patients in drug development for Neglected Tropical Diseases (NTDs): A qualitative study exploring and incorporating preferences of patients with cutaneous leishmaniasis into Target Product Profile development
Source: PLoS Negl Trop Dis. 2024 Feb 21;18(2):e0011975. doi: 10.1371/journal.pntd.0011975 (PMC10965092; doi:10.1371/journal.pntd.0011975)
Supplement: S1 Table — (PDF) [file pntd.0011975.s001.pdf]

Supporting information 1: Overview of study sites and demographic/clinical characteristics of patients enrolled in the study

	Austria	Brazil	Colombia	Colombia	Overall
**Institution/Study site**	Department of Medicine I, Medical University of Vienna, Vienna	Centro de Pesquisa René Rachou (CPqRR), Fundação Oswaldo Cruz (FIOCRUZ) Minas Gerais, Belo Horizonte	Centro Internacional de Entrenamiento de Investigaciones Médicas (CIDEIM), Cali	Programa de Estudio y Control de Enfermedades Tropicales (PECET), Medellín	
**Area where the study is conducted**	Vienna	Belo Horizonte	Cali and Tumaco	Leishmaniasis Recovery Center, Boyacá	
***Leishmania* species**	*L. (Viannia) guyanensis*	*L. (Viannia) braziliensis*	*L. (Viannia) panamensis*	*L. (Viannia) braziliensis, L. (Viannia) panamensis*	L. br. 55%<br>L. gu. 9%<br>L. pa. 36%
**Number of patients**	3	10	10	10	33
**Age range of patients (mean, median) in years**	34-71 (48, 40)	19-71 (42, 41)	18-52 (34, 34)	20-32 (25, 25)	18-71 (35, 32)
**Gender ratio (F:M) in %**	33:66	50:50	20:80	0:100	24:76
**Average number of lesions (mean, median)**	1-2 (1.7, 2)	1-1 (1, 1)	1-4 (1.7, 1)	1-5 (2.4, 1)	1-5 (1.7, 1)
**Treatment status in % (diagnosed:under treatment:completed:untreated)**	0:0:100:0	0:40:60:0	20:40:40:0	20:30:50:0	12:22:55:0

Notes: L.br…. L. *(Viannia) braziliensis*, L.gu…L. *(Viannia) guyanensis*, L.pa….L. *(Viannia) panamensis*
