# Supplementary material for: Involving patients in drug development for Neglected Tropical Diseases (NTDs): A qualitative study exploring and incorporating preferences of patients with cutaneous leishmaniasis into Target Product Profile development
Source: PLoS Negl Trop Dis. 2024 Feb 21;18(2):e0011975. doi: 10.1371/journal.pntd.0011975 (PMC10965092; doi:10.1371/journal.pntd.0011975)
Supplement: S1 File — (PDF) [file pntd.0011975.s002.pdf]

Supporting information 2. Overview of themes by TPP categories

Table 1: Themes related to Safety/tolerability

TPP domain (NVivo top-level node)	TPP Attribute / Category (NVivo node)	Theme
Safety/tolerability	Safety monitoring requirements	Description of types, sequence and timing of tests for monitoring
		Monitoring needs determine treatment location
	Tolerability	Description of AEs
		AEs seen as part of treatment
		Fears about (second) treatment due to AEs
		Preference for lower volume of drug
		Trade-off: Cure vs. tolerability

Table 2: Themes related to Contraindications

TPP domain (NVivo top-level node)	Category (NVivo node)	Theme
Contraindications		Reasons for contraindications
		Alternative therapies
		Self-care
		Shared decision-making

Table 3: Themes related to Efficacy

TPP domain (NVivo top-level node)	Category (NVivo node)	Theme
Efficacy	Complete clinical cure	Wound healing and lesion appearance
		Overall good health and fitness
	Absence of sequelae	Sequels related to the treatment
		Sequels related to the disease
	Improved scar formation	Scar appearance
		Scar as a ‘reminder of the disease’
		Impact on social life
	Disease relapse and reinfection	Risk of reinfection in endemic areas
		Need to repeat treatment
		Parasite clearance
	Importance of cure	Trade-off: Cure vs risks related to treatment
		Trade-off: Cure vs scars/aesthetic results

Table 4: Themes related to Formulation

TPP domain (NVivo top-level node)	Category (NVivo node)	Theme
Formulation	Oral	Ease of use
		Exacerbation of stomach problems
	Parenteral	Fear of needles
		Perceived efficacy of parenteral administration
		Local vs. systemic administration of parenteral treatment

		Trade-off: Pain due to injections vs. perceived disease severity
		Lack of alternatives: Injections as only treatment option
	Topical	Preference for creams
		Thermotherapy
		Topical treatment for a local infection

Table 5: Themes related to Treatment regimen

TPP domain (NVivo top-level node)	Category (NVivo node)	Theme
Treatment regimen	Treatment duration	Optimal treatment duration
		Trade-off: Treatment duration vs. cure
		Administration of therapy outside of treatment facility due to long treatment duration
		Recovery time after treatment
	Compliance	Trade-off: Compliance vs. side effects
		Low compliance due to fear of injections
		Treatment vs. no treatment
		Treatment interruptions due to AEs
	Treatment frequency	Daily vs. weekly administration
		Trade-off: Place of administration vs. treatment frequency

Table 6: Themes related to Target population

TPP domain (NVivo top-level node)	Category (NVivo node)	Theme
Target population	General attitudes in favour of treatment for everyone	Intrinsic right to treatment
		Avoiding disease progression
		Medical instead of traditional treatment
		Treatment after clinical confirmation
	Special populations	Lack of understanding
		No treatment due to strength of medication

Table 7: Themes related to Cost

TPP domain (NVivo top-level node)	Category (NVivo node)	Theme
Cost	Costs of products or procedures	Costs for drugs on black market
		Administration of injections
		Consultation fees
		Costs for inadequate treatment (based on misdiagnosis)
		Costs for diagnostic tests
		Transport costs
	Indirect costs	Profession-related costs (inability to work, or change of occupation)
		Having to stay away from home for treatment

Table 8: Themes related to Perceived barriers

TPP domain (NVivo top-level node)	Category (NVivo node)	Theme
Perceived barriers	Geographical barriers	Distances between home and treatment facility
		Private vs. public transport
		Referral to military treatment facility
		'Being far from home'
	Illegal professions	Guerrillas
		Illicit crops
	Availability of treatment	Treatment within the military treatment facility
		Treatment not authorized by local authorities
	Time to correct diagnosis and start of treatment	Time to correct diagnosis
		Time between positive test and onset of treatment
		Awareness of necessity to seek treatment fast

Table 9: Themes related to Other development needs

TPP domain (NVivo top-level node)	Category (NVivo node)	Theme
Other development needs	Information and dissemination activities	Information about the disease in general
		De-stigmatizing the disease
	Investments in research	Alternative and safer treatments
		Vaccines

	Additional care for patients/supportive therapies	Vitamins to complement therapy
		Care within the military treatment facility
		Physiotherapy
		Psychological counselling
