# Supplementary material for: Involving patients in drug development for Neglected Tropical Diseases (NTDs): A qualitative study exploring and incorporating preferences of patients with cutaneous leishmaniasis into Target Product Profile development
Source: PLoS Negl Trop Dis. 2024 Feb 21;18(2):e0011975. doi: 10.1371/journal.pntd.0011975 (PMC10965092; doi:10.1371/journal.pntd.0011975)
Supplement: S2 File — (PDF) [file pntd.0011975.s003.pdf]

Supporting information 3: Additional findings.

6 Target population

The vast majority of patients asked agreed that everyone should be treated. Among the variety of reasons being given were: An intrinsic right to treatment, and improving patients' quality of life, that otherwise the disease would progress, or in contrast to treatment with home-remedies, or treated after a positive test.

The views of two Colombian patients reflect an intrinsic right to treatment, in order to improve their quality of life, and regardless of where they come from or their financial means:

> *Well, I think that all the people, not because they are from the countryside or because they have money. All should be treated equal. Having a parasite eating your flesh is something no one should have. I do not wish this disease to anyone. I do not know, but I feel that all of us have the right to medical treatment. Regardless if you are from the countryside, poor or rich. Equality for all. I do not wish this illness to anyone. This is ugly. (CC08)*

One patient noted that all persons with CL should be treated, in order to avoid disease progression.

Another one noted that affected persons should be treated, and receive medical treatment, in order to avoid treatment with traditional means, or 'home-remedies'.

> *I would say that a person who is affected by it, who already has the lesions, goes to a center, gets the exams and the treatment, dedicates time to the treatment so it can be effective […]. This disease treated with home-remedies is not going anywhere. (CC09)*

One patient emphasized that everyone with a positive test result, so after clinical confirmation, should be treated.

Patients had differing views on whether special populations, such as children, pregnant women or the elderly should be treated. In contrast to the views reported above as to why everyone should receive treatment, views reflecting why they should not be treated are reported here.

One patient from Brazil reported that they were unsure whether children should be treated, as children had a lack of understanding of the treatment procedure, and its potential toxicity. Another Brazilian patient who had received intravenous pentavalent antimonial, was against treating children, pregnant women and old persons, due to the strength of the treatment.

8 Perceived barriers

Patients perceived a variety of barriers to treatment, including geographical location, availability of treatment, migratory status or occupation (for example, civilian patients from Colombia reported difficult or no access to CL treatment for patients employed in illegal professions, or migrant workers). Often there was a combination of different factors. These barriers were related to indirect costs and delayed initiation of treatment.

Many patients described the time it took for them to come to the treatment facility. In general, Brazilian patients talked about journeys in the range of hours, often on public transport, whereas the Colombian civilian patients reported very long travel durations. The Colombian patients who were with the military in general reported an efficient process of referral to the treatment facility; however, one young soldier complained about the lack of financial support for the trip.

A number of patients discussed private vs. public transport facilities to reach treatment. A Brazilian patient, who was treated along with her son who also suffered from CL, describes the burden being placed on her and her young son due to the journey to the treatment facility, in combination with the injections administered to him:

> *It has been very hard, you know? Especially when I bring my son... Because today is the first time that I come by the health department's car. The other 3 times, I came in my car. Because I had no conditions to wait for the bus provided by my city health system. I had to stay here all day, since 6 AM until I don't know what time, with him. It is not simple to do it with a child. [...] Another day they tried to apply the injection [to my son] and they couldn't... My son was very nervous ... I went home feeling bad. I couldn't go driving. I had to ask for someone to take us. [...] I think that this treatment should be done in my city. I wouldn't have to go to Betim, [another city]. But in my city, there are no trained professional to apply the injection. (BR04)*

> *The health system program of my town provided me the transport because I don't live in the town, I live in a rural area. […] I wish there was the treatment in my town. [...]I wake up at 1 hour of the morning [to come here]. [...] And I come here after six in the morning when the traffic is good. And I'm leaving around 4 PM and getting home around 9:30 PM. [...] Oh, that's tiring. (BR06)*

The theme of great distances between homes and treatment facilities mostly emerged during the interviews of the Colombian civilian patients. One Colombian patient noted that he was treated in Tunja, despite being based in Paipa, about 40 kms away, which resulted in a daily journey for 20 days, leaving at 8:30 am.

> *[The treatment was given] in Tunja. [...] Well, it was not very close because I was there in Paipa, but in the Paipa's unit they did not want to help me, so I was assigned there. [...] The reason given was that they didn't handle minor procedures. (CC01)*

One patient emphasized well the challenges patients from remote areas face, which in turn leads to e.g., seeking alternative therapies:

> *There are parts like the Llanos, Caquetá, […] where getting to a health center or a hospital is very difficult, then many people become infected with that, and there are people who get cured with other methods. (CC09)*

Colombian military patients described their referral to the military treatment facility. In general, patients reported having to make their way on their own, after being told where to go. Only one patient noted difficulties and expenses involved (*Indirect costs, Transport costs* in the TPP category *7 Costs*).

One Colombian patient describes a general sense of fear experienced by inhabitants of remote regions, being 'far from home', combining a fear of having nowhere to stay, no knowledge of the city, meeting HCPs, and injections.

> *But there are people who live in a very remote area and have nowhere to arrive here, neither knowledge of the city, nothing like that… they dread the city and many things […]. All that makes it worse, right? And some might say that they are afraid of doctors and so many injections... and live there with the disease. (CC09)*

On several occasions, civilian patients from Colombia reported difficult or no access to CL treatment for patients employed in illegal professions, or migrant workers. One patient describes a rumour related to guerrillas, who are not able to receive treatment through the official HC system:

Here it is completely different, there are people who do not receive any treatment [such] as those in the guerrillas. […] I do not know if they can be cured or not, but that's what I heard. (CC02)

Others discussed the situation of illicit agricultural workers planting or harvesting (‘*raspachines*’) drugs or working as manual eradicators (as an alternative to glyphosate), contracting CL during their work and move before completing or accessing treatment. One Colombian patient tells his story about how they were infected.

Work yes, well, I couldn’t work. [...] No, [I do] not really [want to go back to the work I was doing before]. God willing, I will become well and well, I will go to [my hometown …] I used to work in the fields cultivating greens and all that, and as it became very dry, I had to come here. […) I was working here, as they say, in illicit work. And it was here that I got the disease. (CC07)

In general, those patients in Colombia who were members of the military were very happy about their treatment within the military treatment facility, and one soldier noted that more facilities like this should be established in other parts of the country:

Well, there they treated me very nice in order to the treatment to work properly. Everything I needed was there. [...] Everyone [helps]. You don’t have to worry about the cleaning or something like that. If the soldier is very bad, then auxiliary soldiers bring him food and stuff. (CP10)

The attention they give here it's very good. So, it would be necessary to build more places like this in other parts of the country. (CP02)

In contrast to this were experiences like this patient from Austria, who had to convince a health insurance officer that he contracted the disease on a research trip related to his job, in order to obtain miltefosine, a treatment not authorized by the local authorities for CL. In Austria, miltefosine is only authorised as a cancer drug.

The other possibility, miltefosine, was not authorized in Austria, that is, not for leishmaniasis [...]. Also, it needed to be approved by the health insurance. So, I had to go to another pharmacy in order to obtain the prescription, then to come back for approval, then to the health insurance. There it was funny: I arrived and came to see someone, and said ‘tropical disease leishmaniasis’. The lady attacked me: ‘Ah, again such a tourist who goes somewhere, and we can pay for it...’ [I said] 'Sorry, no, that was a research trip, that’s my job.’ Ok, then she checked the computer, what would I like? Why don’t I take the cheap one? And I explained again: ‘No, I have to leave for Costa Rica in 3 days...’ [She went on:] ‘There you also need to go?’ Then I said: ‘No sorry, that’s also a business trip.’ Then I really checked with her the course directory of the university – look, that’s an occupational accident [...], and also the treatment preference. Then she said: ‘OK, here you go’, and then I started treatment. (AT04)

In general, many patients reported a long time to correct diagnosis, mostly due to a lack of experience on the side of HCPs.

Several Brazilian patients described consulting several doctors before a correct diagnosis was made.

Health professionals do not know the disease. […] There were three doctors who didn't recognize the disease. [...] It would not have reached that size ... my wound was small at the beginning, and there are people who get worse, worse, worse. (BR01)

Doctors should have more knowledge about this disease, because, in Rio Manso, there are other cases. (BR04)

A number of patients reported being wrongly prescribed medication, such as antibiotics or cortisone, exacerbating the lesion.

Some Colombian soldiers also talked about their efforts to receive medical attention due to CL.

> *I had to beg at the battalion to get out. [...] From the area to the battalion, [it took me] 1 month and 20 days. I was under training with that wound. I had to do it. […] I tried to protect it as much as I could from the mud when they asked us to crawl. (CP09)*

Patients describe different views on the time between a positive test and the start of treatment. Two patients, members of the Colombian military, describe receiving treatment the next day, whereas one Colombian civilian patient describes how approval of treatment after diagnosis took about 15 days, during which the lesion grew significantly.

> *[Getting a diagnosis] was, in my point of view, simple. [...] The approval of the treatment was what took long. […] There are people who were ordered the treatment, and they have waited for months in the area, because they were from there, can you imagine how bad can they get in just one month. (CC01)*

In general, many patients were aware of the necessity to seek and start treatment quickly in order to avoid sequels, such as disfigurement, or death.

> *I was told to move quickly because if that went up to the blood it could end my life [...] [I was told] to look for a doctor, and fast, because that could mess my face up pretty bad. That my sight could start to fail. (CC02)*

> *I think so, yes [it's important to look for help fast]. [...]. [The treatment is going to be easier]. [...] It's not [an option to not receive the treatment]. It will get every time worse if you don't treat the disease. [...] I think, if the disease develops too much, you can die. (CP06)*
